# Modeling and Structural Optimization of MSF-RO Desalination System

**DOI:** 10.3390/membranes12060545

**Published:** 2022-05-24

**Authors:** Lu He, Aipeng Jiang, Qiuyun Huang, Yan Zhao, Chuang Li, Jian Wang, Yudong Xia

**Affiliations:** 1 School of Automation, Hangzhou Dianzi University, Hangzhou 310018, China; 202060285@hdu.edu.cn (L.H.); hqy09092020@163.com (Q.H.); lichuang_1006@163.com (C.L.); 02016@hdu.edu.cn (J.W.); 42072@hdu.edu.cn (Y.X.); 2 Chn Energy Lucency Enviro-Tech Co., Ltd., Beijing 100039, China; 12030975@chnenergy.com.cn

**Keywords:** desalination, MSF, RO, operational cost, modeling, simulation, structural optimization

## Abstract

Based on the mathematical modeling and operational optimization studies of reverse osmosis (RO) and multistage flash (MSF) desalination, the structural optimization of the hybrid process was specially studied in this work with the consideration of reducing comprehensive expenses under given operational conditions. Firstly, according to the process mechanism and flowchart of the RO and MSF seawater desalination technologies, seven hybrid structures with different feed conditions were designed, and their connection equations were established for numerical calculation. Then, in order to evaluate the economic performance of the hybrid systems with different structures, the hourly average operational cost equations of RO and MSF processes were established and formulated as the comprehensive evaluation indicators. Next, with a given water production requirement, simulation calculations of the hybrid system with seven different structures were performed. The results show that the hybrid system with the fourth structure has the lowest operational cost of 4.6834 CNY/m^3^, and at the same time it has the lowest blowdown. However, if we take GOR or production water temperature as the target, the optimal structure of the hybrid system is the fifth or the seventh option. The obtained results are helpful in structural optimization of the hybrid system with aspects of operational cost reduction, maximum GOR, or minimizing the wastewater discharge.

## 1. Introduction

The lack of fresh water resources is an indisputable fact, and desalination technology is an effective strategy to solve this problem [1,2]. At present, among many seawater desalination technologies, multistage flash (MSF), and reverse osmosis seawater (SWRO) desalination are the most effectively and widely technologies used in practice. However, with the increased requirement of cost-saving and wastewater discharge reduction, new challenges are presented to improve seawater desalination process [3]. Since the MSF technique [4,5] can get a large amount of higher quality fresh water from low-grade energy and its concentration is significantly lower than that of the SWRO technique, the good complementary characteristics of these two techniques become an attractive direction to improve the overall profits for freshwater achievement with lower operational costs. At present, the MSF technology and SWRO technology are relatively mature, and there are some studies about the hybrid thermal-membrane desalination technology, but the detailed research on the optimal structure of the hybrid system is relatively novel and deficient [6]. Since both the SWRO desalination and the MSF desalination are energy-intensive technologies, most new studies are focused on the couple of desalination technique with the energy-driven system. For example, Khan et al. [7,8] carried out fruitful work on the desalination system coupled with nuclear power. In their studies, a techno-economic analysis of a nuclear power plant coupled with RO or MED or RO + MED and even RO + MSF systems were assessed in detail. With the consideration of environmental impact, they also built a simulation model for calculation and assessment of the socio-economic and environmental impacts of a nuclear desalination system.

In many regions where there are no renewable energies or nuclear energy available, the hybrid desalination technologies for full utilization of energy and cost saving were also studied by researchers. Kuenstle et al. [9] proposed a design concept for a combined MSF-RO and power plant system. They evaluated its economic benefits, and concluded that the combined system has lower water production costs than a single MSF system. Al-marafie [10] carried out the economic comparison between MSF and MSF-RO seawater desalination systems—the results showed that the MSF-RO system has more advantageous than MSF. In addition, the author proposed that replacement of the local traditional desalination system with the MSF-RO system has better application prospects. Awerbuch et al. [11] studied the SWRO desalination system and MSF-RO hybrid seawater desalination system, and designed two MSF-RO and one MSF-RO-VEC seawater desalination systems with different coupling structures. In the same year, Awerbuch et al. [12] designed a hybrid system, analyzed the characteristics of the MSF desalination system and RO desalination system, and after 1987, the authors still affirmed the advantages of the MSF-RO seawater desalination system and gave a structure diagram of the initially designed hybrid system. In the same year, Al-Mutaz et al. [13] designed a relatively simple MSF-RO system and established the system model. However, it has not been verified and simulated, and the subsequent system optimization work has not been carried out. El-Sayed et al. [14] designed the MSF-RO experimental device and analyzed and studied the experimental data. After 1800 h of experimental testing, the experimental data proved the superiority of the MSF-RO system in terms of desalination rate, fresh water output, and energy conservation. However, this conclusion can only be regarded as a qualitative background, not enough to draw a specific conclusion, and further research is needed. However, at least based on our literature knowledge, the above work did not carry detailed model-based numerical analysis on the optimal design and operation of systems. A reasonable and accurate mathematical model, which can fully describe the important process parameters and their influences, is very important for the study of the subsequent system. Most of the above research work affirms the superiority of the MSF-RO system, but at least based on our literature knowledge, the above work does not carry out detailed model-based numerical analysis for optimal system design and operation.

For the detailed quantitative analysis and performance prediction requirement to guide the hybrid system’s application, a series of modeling, simulation, and optimization works have been carried out at home and abroad. The relevant characteristics of the hot-film hybrid system is deeply understood by using the calculation tool. Cardona et al. [15] studied the changes in energy saving and water production cost after the integration of the MSF system and RO system. Based on the established water production model, the levels of energy consumption and water production before and after the system integration were obtained, which emphasized the advantages of MSF-RO in energy saving. Helal et al. [16,17,18] established seven different coupling forms of the MSF-RO seawater desalination system, in addition to a two-stage SWRO seawater desalination system model and a BR-MSF seawater desalination model. Finally, various schemes are compared and evaluated, and it is concluded that through integrating MSF with the SWRO technique, the water production cost of the system can be reduced by 17% to 24%. Marcovecchio et al. [19] established a simple hybrid MSF-RO desalination system with reference to the work of Helal and others, taking the highest daily fresh water output as the optimization goal and solving it. The MSF part of the model was the OT-MSF seawater desalination system, which had a simple structure. Only the discharge of the MSF system and the feed of the RO system are coupled, and the coupling mode is relatively single. Vince et al. [20] established a super-model of the MSF-RO seawater desalination system, and proposed a method of optimization problem decomposition and realization of multiobjective optimization. This method combined process modeling and process integration technology with advanced mathematical solving tools to simultaneously optimize the configuration and operation of the integrated desalination system. Moreover, this method has been verified in the model established in this paper. Abdulrahim et al. [21] used the first and second laws of thermodynamics to conduct a rigorous modeling and simulation study on the hybrid MSF-RO system. The results showed that the performance of the MSF-RO integrated system had been enhanced. The study considered four optimization goals, namely the maximum fresh water production, the minimum water production cost, the maximum gain ratio, and the minimum exergy damage rate; and the genetic algorithm was used to optimize the multiobjectives to make this system meet the requirements.

Wu et al. [22] adopted the concept of hybrid nodes and distribution nodes, and established a superstructure model of the MSF and RO seawater desalination system. Regarding the water production ratio as an optimization variable of the integrated system, an improved genetic algorithm was used to get the optimal solution. Malik et al. [23] used the Aspen custom modeling tool (ACM, V8.4.) to model and simulate MSF, RO, and MSF-RO respectively. The author took the lowest economic objective function as the optimization goal, and optimized the system operating conditions and process design variables. However, in his study, only one simple MSF-RO coupled structure was considered. For application intention, Boushi [24] studied two system transformation schemes for the current status of the MSF plant in Al Taweelah A2 in Abu Dhabi, UAE. Based on practical data and the performance model of MSF, RO, and MSF-RO systems, the techno-economics and environmental impact were analyzed. To realize detailed numerical calculations and global optimization for a more complex system, Bandi et al. [25] used differential evolution algorithm (DE) to globally optimize the MSF-RO desalination system with five different MSF-RO alternative mixing schemes. The results showed that the DE algorithm was suitable for the solving optimization problems of the more complex MSF-RO system. The given system and algorithm can be used to estimate the flowrate, the salt content of blowdown brine, circulating brine flowrate, the salt content, and condensate flowrate.

The above studies have confirmed the advantages and prospects of the MSF-RO system, and carried out relevant work in the aspect of optimal control. However, there are still some works that should be done for better guiding the structural and operational optimization of the MSF-RO system, especially in the plants who need to upgrade their RO or MSF system and achievement better operational profits. In this work, based on our earlier studies of full-scale detailed mechanism models of SWRO and MSF systems, detailed structural optimizations of the MSF-RO system under certain operational conditions and with techno-economics indexes are studied. After establishing the complete mechanism models of MSF and SWRO systems, according to the different feeding, mixture, and separation forms of the MSF-RO system, seven structural combination forms are designed and modeled for numerical calculation, and furthermore, the economic and performance equations are established for comparative analysis end scheme evaluation. Our work is helpful to guide structural optimization and detailed quantitative analysis of the MSF-RO system.

## 2. Different Structures of Hybrid MSF-RO System and Their Connection Equations

In order to deeply analyze the impact of its feeding mode on the economy of the hybrid MSF-RO seawater desalination system, this paper proposes seven hybrid schemes of the MSF-RO system according to different feeding conditions:

1. The OT-MSF system and SWRO system feed water independently;
2. The BR-MSF system and SWRO system feed water independently;
3. The last stage flash water of the OT-MSF system is the feed water of the SWRO system;
4. The last stage flash water of the BR-MSF system is the feed water of the SWRO system;
5. The brine in the thermal discharge section of the BR-MSF system is used as the feed water of the SWRO system;
6. The rejected water of the SWRO system is used as part of the feed water of the OT-MSF system;
7. The rejected water of the SWRO system is used as part of the feed water of the BR-MSF system.

In order to facilitate the analysis and make the study more targeted, in this work, the MSF desalination adopted 16-stage OT-MSF and BR-MSF systems, respectively. The SWRO system adopted the SW30HR-400i spiral-wound RO modules, and put seven RO modules in each pressure vessel. The whole system depends on the actual situation to determine the number of pressure vessels. Since the mechanism models of MSF process and SWRO process have been established by our earlier work and can be referred in literatures, the model equations of the MSF and SWRO system that were used in our work were not listed in this paper. The steady-state models of OT-MSF and BR-MSF systems can be seen from Mujtaba [26,27,28], Woldai A [29], Rosso [30], and Gao [31] et al. The steady-state mechanism model of the SWRO system can be seen from Jiang [32,33,34], Ma [35], and Cheng [36] et al. This paper just shows the different schemes of the hybrid system and the equations to connect the MSF and RO processes, and this is enough to achieve the whole model of the hybrid system under a given coupling structure.

### 2.1. Hybrid System with Independent Feeding

#### 2.1.1. Scheme of OT-MSF and SWRO System with Independent Feed Water

**Figure 1:**
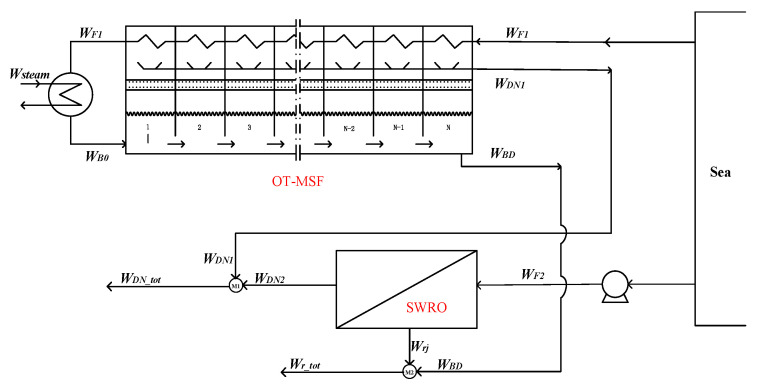
Option 1—the OT-MSF system and SWRO system with independent feed water.

As is shown in the Figure 1. In the option 1, the hybrid system is composed by OT-MSF and SWRO parts with independent feed water—that is to say, the feed conditions of the two parts are the same, and they are operated almost completely independently of each other.

Mixer M_1_:

(1) $$W_{DN\_tot}=W_{DN1}+W_{DN2}$$

Mixer M_2_:

(2) $$W_{r\_tot}=W_{rj}+W_{BD}$$

The hybrid option 1 only affects the water production temperature and concentration of the coupling system, and has no effects on the overall water production of the system. It can be used as the basic scheme for hybrid scheme comparison.

#### 2.1.2. The BR-MSF System and SWRO System with Independent Feed Water

**Figure 2:**
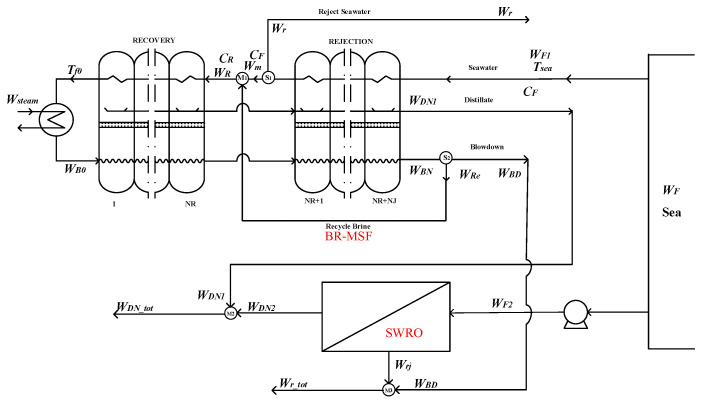
Option 2—the BR-MSF system and SWRO system with independent feed water.

As is shown in the Figure 2. The hybrid system is composed by BR-MSF and SWRO parts with independent feed water.

Splitter S_1_:

(3) $$W_{m}=W_{F}-W_{r}$$

Splitter S_2_:

(4) $$W_{BD}=W_{BN}-W_{Re}$$

Mixer M_1_:

(5) $$W_{R}=W_{m}+W_{Re}$$

Mixer M_2_:

(6) $$W_{DN\_tot}=W_{DN1}+W_{DN2}$$

Mixer M_3_:

(7) $$W_{r\_tot}=W_{rj}+W_{BD}$$

In the hybrid option 2, the splitters S_1_, S_2_, and the mixer M_1_ are self-owned by the BR-MSF system. As in hybrid option 1, option 2 only couples the system water production and brine water, which has an impact on the water production temperature and concentration of the hybrid system, but has no impact on the overall water production of the system.

### 2.2. The Last Stage Flash Water of the MSF System as the Feed Water of the SWRO System

#### 2.2.1. The Last Stage Flash Water of the OT-MSF System as the Feed Water of the SWRO System

**Figure 3:**
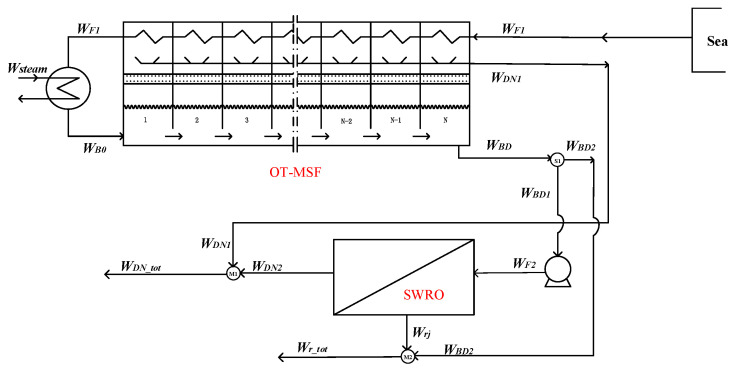
Option 3—the last-stage flash water of the OT-MSF system as the feed water of the SWRO system.

In the hybrid option 3, part of the blowdown water *W_BD_*_1_ in the OT-MSF system is used as the feed water *W_F_*_2_ of the SWRO system, removing the seawater pretreatment link in the SWRO system. Its flow chart is shown in Figure 3. The whole coupling system summarizes the water production of the OT-MSF system and the SWRO system. At this time, the overall water production and temperature are affected.

Splitter S_1_:

(8) $$W_{BD1}=W_{BD}-W_{BD2}$$

Mixer M_1_:

(9) $$W_{DN\_total}=W_{DN1}+W_{DN2}$$

Mixer M_2_:

(10) $$W_{r\_tot}=W_{rj}+W_{BD2}$$

#### 2.2.2. The Last-Stage Flash Water of the BR-MSF System as the Feed Water of the SWRO System

**Figure 4:**
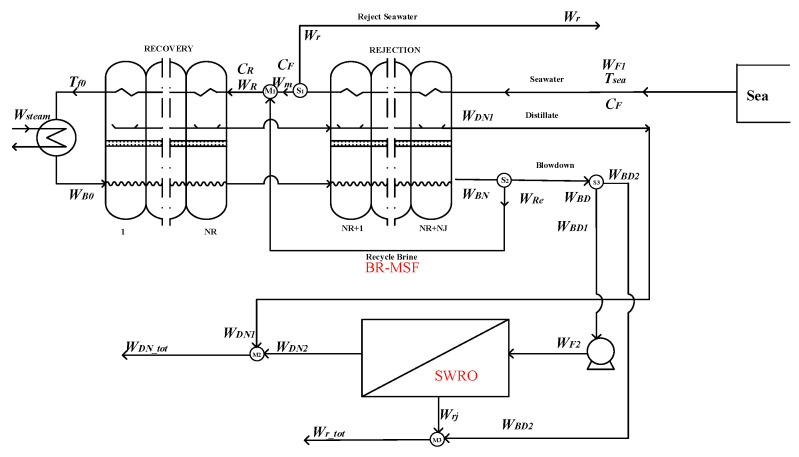
Option 4—the last-stage flash water of the BR-MSF system as the feed water of the SWRO system.

Similarly to the hybrid situation of option 3, in option 4, part of the blowdown water *W*_*BD*1_ in the BR-MSF system is used as the feed water *W_F_*_2_ of the SWRO system, and the seawater pretreatment in the SWRO system is removed. Its flow chart can be seen in the Figure 4. The whole hybrid system summarizes the water production of the BR-MSF system and the SWRO system. At this time, the overall water production and temperature are affected.

Splitter S_1_:

(11) $$W_{m}=W_{F1}-W_{r}$$

Splitter S_2_:

(12) $$W_{BD}=W_{BN}-W_{Re}$$

Splitter S_3_:

(13) $$W_{F2}=W_{BD}-W_{BD2}$$

Mixer M_1_:

(14) $$W_{R}=W_{m}+W_{Re}$$

Mixer M_2_:

(15) $$W_{DN\_tot}=W_{DN1}+W_{DN2}$$

Mixer M_3_:

(16) $$W_{r\_tot}=W_{rj}+W_{BD2}$$

### 2.3. The Rejected Water of the BR-MSF System as the Feed Water of the SWRO System

**Figure 5:**
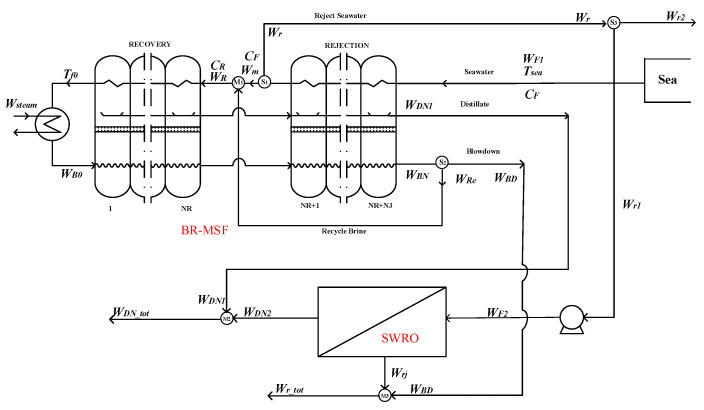
Option 5—the rejected water of BR-MSF system as the feed water of the SWRO system.

The process flow diagram can be seen in the Figure 5. In option 5, part of the rejected water *W*_*r*1_ of the BR-MSF system is used as the feed water *W_F_*_2_ of the SWRO system. At this time, the temperature of the feed water in the SWRO system is increased, and the whole process of seawater pretreatment is removed. At this time, the entire hybrid system aggregates the production water and wastewater of the two subsystems. At this time, the overall water production and quality of the system are affected.

Splitter S_1_:

(17) $$W_{m}=W_{F1}-W_{r}$$

Splitter S_2_:

(18) $$W_{BD}=W_{BN}-W_{Re}$$

Splitter S_3_:

(19) $$W_{F2}=W_{r}-W_{r2}$$

Mixer M_1_:

(20) $$W_{R}=W_{m}+W_{Re}$$

Mixer M_2_:

(21) $$W_{DN\_tot}=W_{DN1}+W_{DN2}$$

Mixer M_3_:

(22) $$W_{r\_tot}=W_{rj}+W_{BD2}$$

### 2.4. The Rejected Water of the SWRO System as Part of the Feed Water of the MSF System

#### 2.4.1. The Rejected Water of the SWRO System as Part of the Feed Water of the OT-MSF System

Among the five options mentioned above, the output of the MSF system is used as the feed water of the SWRO system. Options 6 and 7 are compared, on the other hand, with the wastewater from the SWRO system and part of the seawater as the feed water for the MSF system.

**Figure 6:**
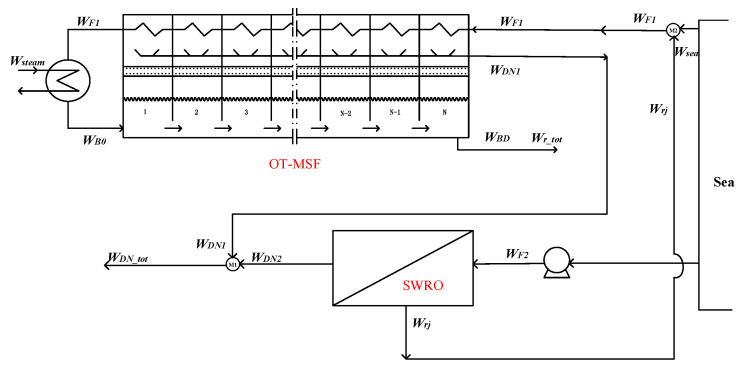
Option 6—the rejected water of the SWRO system as part of the feed water of the OT-MSF system.

In option 6, the blowdown water *W_rj_* of the SWRO system is used as the feed water *W_F_*_1_ of the OT-MSF system. Figure 6 shows the flow of this option. In this case, the feeding water conditions of the OT-MSF system are changed, and the overall water production of the hybrid system also changes. The hybrid system combines the water production of the two systems, and the blowdown water of the hybrid system is only discharged from the OT-MSF system.

Coupling system blowdown:

(23) $$W_{r\_tot}=W_{BD}$$

Mixer M_1_:

(24) $$W_{DN\_tot}=W_{DN1}+W_{DN2}$$

Mixer M_2_:

(25) $$W_{F1}=W_{rj}+W_{sea}$$

#### 2.4.2. The Rejected Water of the SWRO System as Part of the Feed Water of the BR-MSF System

**Figure 7:**
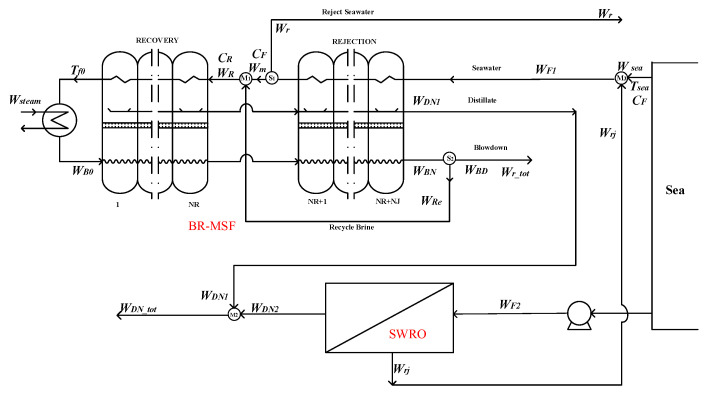
Option 7—the rejected water of the SWRO system as part of the feed water of BR-MSF system.

In option 7, the blowdown water *W_rj_* of the SWRO system is used as the feed water *W_F_*_1_ of the BR-MSF system. Figure 7 shows the flow of this option. At this time, the feeding water conditions of the BR-MSF system are changed, and the overall water production of the coupled system also changes. The hybrid system combines the water production of the two systems, and the blowdown water of the hybrid system is only discharged from the BR-MSF system.

Coupling system blowdown:

(26) $$W_{r\_tot}=W_{BD}$$

Splitter S_1_:

(27) $$W_{m}=W_{F1}-W_{r}$$

Splitter S_2_:

(28) $$W_{BD}=W_{BN}-W_{Re}$$

Mixer M_1_:

(29) $$W_{R}=W_{m}+W_{Re}$$

Mixer M_2_:

(30) $$W_{DN\_tot}=W_{DN1}+W_{DN2}$$

Mixer M_3_:

(31) $$W_{F1}=W_{rj}+W_{sea}$$

## 3. Operation Economics and Evaluation Index of the MSF-RO System

### 3.1. Operational Economic Model of the SWRO System

The economic model discussed in this article is an hourly operating cost model, and the investment cost is ignored due to the unequal years of plant service life. The operation cost of the SWRO system mainly includes the following seven parts:

1. Preliminary energy consumption cost *OC_IP_*;
2. Chemical cost *OC_CH_*;
3. Operating energy consumption cost *OC_EN_*;
4. Reverse osmosis membrane replacement cost *OC_MER_*;
5. Maintenance cost *OC_MN_*;
6. Labor costs *OC_LB_*_1_;
7. Wastewater management cost *OC_WS_*_1_.

The relevant expression of the corresponding operation cost of each part is as follows:

*OC_IP_*:

(32) $$OC_{IP}=\frac{P_{in}\cdot W_{F2}\cdot P_{elc}}{\eta_{IP}}\times PLF$$

The preliminary energy consumption cost of SWRO system refers to the energy consumption cost of water intake and pretreatment. *P_in_* refers to the outlet pressure of the water intake pump, which is 5 bar; *W_F_*_2_ is the feed water flowrate; *P_elc_* is the price of electricity; *PLF* is the load factor, which is 0.9; $\eta_{IP}$ is the motor efficiency, which is 0.85.

*OC_CH_*:

(33) $$OC_{CH}=0.235W_{F2}$$

The cost of chemical agents mainly includes various acid reagents, scale inhibitors, flocculants, and other additives that change the water hardness.

*OC_EN_*:

(34) $$OC_{EN}=\left[P_{f}\cdot W_{F2}/\left(\eta_{hp}\cdot \eta_{fd}\right)-P_{r}\cdot W_{rj}\cdot \eta_{bp}\right]\cdot P_{elc}$$

where *P_f_* denotes the feed water pressure, which is 65 bar; $\eta_{hp}$ refers to the high-pressure pump efficiency, which is 0.85; $\eta_{fd}\;$ is the mechanical efficiency of the variable frequency drive, which is 0.94; *P_r_* is the outlet pressure of concentrated brine; *W_rj_* refers to the concentrated brine outlet flowrate; $\eta_{bp}$ is the booster pump mechanical efficiency, which is 0.8.

*OC_MER_*:

(35) $$OC_{MER}=Pri_{ME}\cdot MOD\cdot \zeta_{re}/365/24$$

where *Pri_ME_* is the unit price of the RO membrane, 6200 CNY/group; *MOD* refers to the total number of RO membranes used in the system; $\zeta_{re}$ represents the replacement rate of membrane modules, which is 0.3.

*OC_MN_*:

(36) $$OC_{MN}=OC_{MNCON}+OC_{MNCL}$$

(37) $$OC_{MNCL}=Ncl\cdot \left(OC_{OT}+OC_{PC}\right)/Xmr$$

The maintenance cost of the SWRO system is composed of the maintenance cost of conventional equipment *OC_MNCON_* and the cleaning and maintenance cost of reverse osmosis membrane module *OC_MNCL_*. *N_cl_* is the number of membrane module cleanings in a membrane replacement cycle; *X_mr_* is the membrane replacement cycle; *OC_OT_* is the cost of chemicals in membrane cleaning; *OC_PC_* is the system startup and shutdown costs incurred by the cleaning operation.

*OC_LB_*_1_:

(38) $$OC_{LB1}=Pri_{LB}\cdot N_{LB}/24$$

(39) $$N_{LB}=W_{DN2}\cdot NP/100$$

*N_LB_* refers to the number of labors. *W_DN_*_2_ represents the water production of the SWRO system, and *NP* refers to the number of pressure vessels in the system.

*OC_WS_*_2_:

(40) $$OC_{WS2}=0.025\cdot W_{rj}\cdot NP$$

where *W_rj_* is the blowdown water from the SWRO system.

This article studies the operation plan of the system within the day, and the cost of RO membrane cleaning can be ignored. Therefore, Equation (36) can be rewritten as Equation (41) as shown:

(41) $$OC_{MN}=OC_{MNCON}=0.03OC_{RO}$$

The total cost of SWRO system can be formulated as follows:

(42) $$\begin{array}{ll} OC_{RO} & =OC_{IP}+OC_{EN}+OC_{MER}+OC_{MN}+OC_{CH}+OC_{LB1}+OC_{WS1}\\ & =OC_{IP}+OC_{EN}+OC_{MER}+OC_{MNCON}+OC_{CH}+OC_{LB1}+OC_{WS1}\\ & =OC_{IP}+OC_{EN}+OC_{MER}+0.03OC_{RO}+OC_{CH}+OC_{LB1}+OC_{WS1}\\ & =\left(OC_{IP}+OC_{EN}+OC_{MER}+OC_{CH}+OC_{LB1}+OC_{WS1}\right)/0.97 \end{array}$$

### 3.2. Operational Economic Model of MSF System

Likewise, the economic model of the MSF seawater desalination system is only for the hourly operating cost model. It mainly includes the following six parts, namely:

1. Heating steam, *OC_ST_*;
2. System power consumption cost, *OC_EL_*;
3. Maintenance cost, *OC_MT_*;
4. Pretreatment cost, *OC_PR_*;
5. Maintenance cost, *OC_MN_*;
6. Labor costs, *OC_LB_*_2_;
7. Wastewater management cost, *OC_WS_*_2_;

The relevant expression of the corresponding operation cost of each part is as follows:

*OC_ST_*:

(43) $$OC_{ST}=22/24\cdot W_{steam}\cdot \left[\left(T_{steam}-40\right)/85\right]\cdot 0.00415\cdot 6.4525\cdot 1.45$$

The MSF system economic model can be seen from Gao et al. [31], and the corresponding reference is the data in the paper by Wade et al. [37] in 2001. Taking into account the US dollar inflation, the GDP deflator is used for conversion. Areppim website provides a dollar conversion calculator for each year based on the GDP deflator, and the dollar in 2001 is equivalent to $1.45 in 2021. Based on the exchange rate between USD and RMB at 6.4525, the economic model of MSF seawater desalination system is synthesized, as shown in the following formulas.

*OC_EL_*:

(44) $$OC_{EL}=22/24\cdot \left(W_{DN}/\rho_{w}+0.005\cdot W_{F}/\rho_{b}\right)\cdot \left(P_{elc}/3\right)\cdot 6.4525\cdot 1.45$$

*OC_MT_*:

(45) $$OC_{MT}=22/24\cdot \left(W_{DN}/\rho_{w}\right)\cdot 0.082\cdot 6.4525\cdot 1.45$$

*OC_PR_*:

(46) $$OC_{PR}=22/24\cdot \left(0.15\cdot W_{F}/\rho_{b}+0.1\cdot W_{DN}/\rho_{w}\right)\cdot 0.024\cdot 6.4525\cdot 1.45$$

*OC_LB_*_2_:

(47) $$OC_{LB2}=22/24\cdot \left(W_{DN}/\rho_{w}+0.005\cdot W_{F}/\rho_{b}\right)\cdot 0.1\cdot 6.4525\cdot 1.45$$

*OC_WS_*_2_:

(48) $$OC_{WS2}=0.025\cdot \left(W_{BD}/\rho_{b}\right)$$

The specific cost of the above six parts, which make up the total hourly operational costs of the MSF system can be expressed as:

(49) $$OC_{MSF}=OC_{ST}+OC_{EL}+OC_{MT}+OC_{PR}+OC_{LB2}+OC_{WS2}$$

### 3.3. Evaluation Indicators and Economic Model Verification

The economic model of MSF-RO system is composed of the economic model of MSF system and SWRO system. In order to evaluate the economy of the hybrid model, the economic models of the OT-MSF system and the SWRO system need to be verified respectively. For the MSF system, the OT-MSF system is selected for economic model verification. This study only discusses the case that the seawater temperature is 25 °C and the electricity price is 0.67 CNY/KW h. The verification premises of the economic model of MSF-RO hybrid system are as follows:

1. The feed seawater flowrate of the OT-MSF system and the SWRO system are both 1.203 × 10^7^ kg/h = 1.203 × 10^4^ m^3^/h.
2. The heating steam temperature is 97 °C.
3. The feed seawater concentration of the OT-MSF system is 5.7% = 57 kg/m^3^, and the feed seawater concentration of the SWRO system is 30 kg/m^3^. Because the pretreatment requirements of the system are inconsistent, the feed seawater concentrations of MSF system and RO system are different.
4. The unit price of single RO membrane of SWRO system is 6200 CNY.

**Figure 8:**
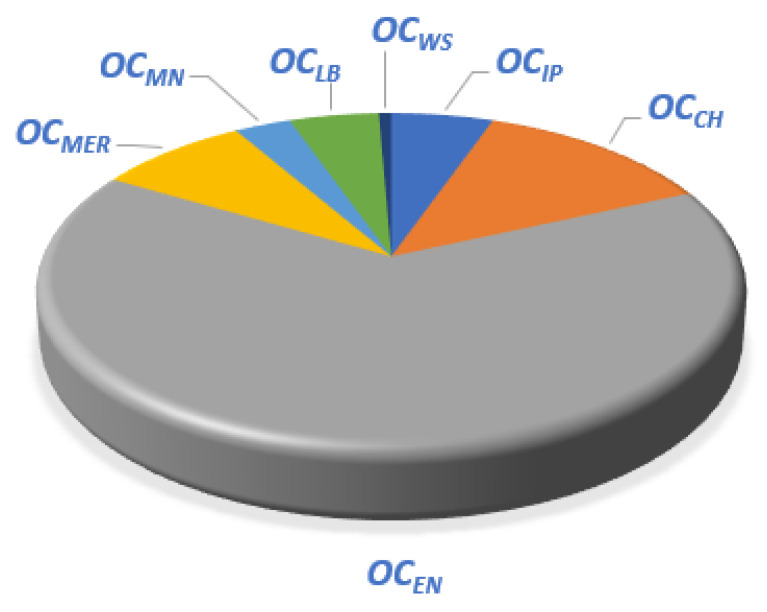
SWRO cost proportion analysis.

Substitute the above parameters into the SWRO system economic model, and the IPOPT and CONOPT solver of the GAMS platform is called for simulation [38]. The SWRO system water production and system cost ratio are shown in Table 1 and Table 2. And the cost analysis is shown in Figure 8.

**Figure 9:**
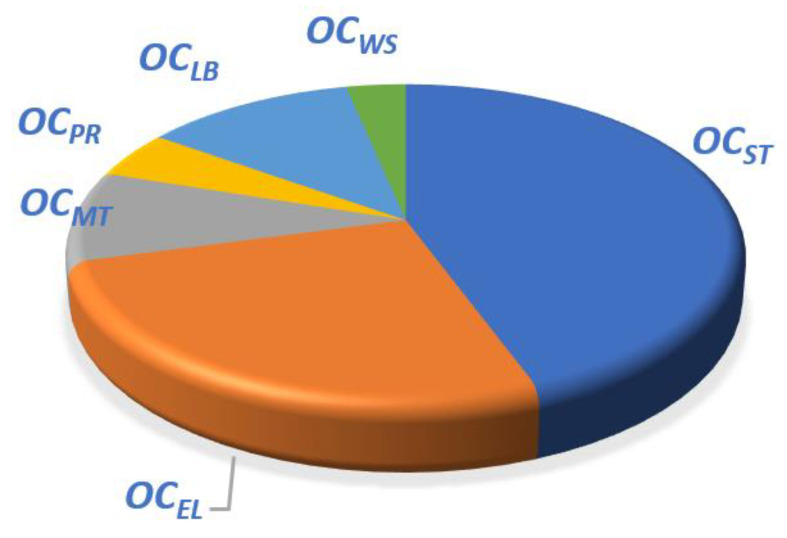
OT-MSF cost proportion analysis.

The above parameters were substituted into the OT-MSF system economic model, and simulated on the GAMS platform. The OT-MSF system water production and system cost ratio are shown in Table 3 and Table 4. The cost analysis is shown in Figure 9.

Under the same water intake flowrate conditions, the total price and cost ratio of the water production cost of SWRO and MSF systems can be obtained, respectively. For the SWRO system, the unit price of water production is 3.51 CNY/m^3^, and for the OT-MSF system, the unit price of water production is 7.63 CNY/m^3^. The ratio of the MSF system to the SWRO system’s water price is 0.4594, and Fan [39] et al.’s statistical result ratio is 0.4600. Therefore, it is proved that the economic model designed in this study can reflect the economic situation of the system and it can be used for analysis and research. It can be seen from Figure 8 that in the cost of SWRO, the *OC_EN_* accounts for the highest proportion of the overall cost, exceeding 50% of the overall cost, and the remaining costs account for a small proportion. It can be seen from Figure 9 that among the OT-MSF costs, the *OC_ST_* accounts for the highest proportion of the overall cost, followed by the system power consumption cost, and the remaining costs account for a relatively low proportion. On the whole, the operation cost of SWRO and MSF system is 1:2, and both account for the highest proportion in the power consumption of the two processes.

## 4. Economic Evaluation of MSF-RO System

In order to quantitatively evaluate the economy and water production performance of the MSF-RO system with different feeding modes, it is necessary to fix the water production and water production ratio of each option. According to the research of Helal et al. [16], assuming that the actual demand ratio of the hybrid seawater desalination plant is 1:2, the MSF system of each hybrid system in this study stipulates that the water production is 1000 m^3^/h, the SWRO system stipulates that the water production is 2000 m^3^/h. The relationship between the water production flowrate of the two systems is 1:2, and the overall water production flowrate is 3000 m^3^/h. The detailed model of the coupled system with different structures are coded and implemented under the platform of GAMS 24.0, and the IPOPT was used as the solver to solve the complex nonlinear programming problems.

### 4.1. Simulation of the MSF-RO System with Different Structures

#### 4.1.1. The OT-MSF System and SWRO System with Independent Feed Water

The simulation results are shown in Table 5 and Table 6. The water production of the hybrid system is shown in Table 7. In this option, the total operating cost of the SWRO system is 7012.21 CNY, of which the running energy consumption accounts for 65.13%. The unit price of SWRO system water production is 3.5061 CNY/m^3^. For the OT-MSF system, the total operating cost is 7422.27 CNY, of which the running energy consumption accounts for 27.21%. Additionally, the unit price of OT-MSF water production is 7.4223 CNY/m^3^. Both systems account for a large proportion of energy consumption costs.

It can be seen from Table 7 that the water production ratio of the SWRO system is relatively high, at 0.52. The water production ratio of the MSF system is 0.092, and the water production ratio of the MSF-RO system is 0.2. It can be seen that the seawater utilization rate of the MSF system is low, and the water production ratio of the MSF-RO hybrid system is improved compared with the MSF system. At the same time, compared with the MSF system, the MSF-RO hybrid system reduces the unit price. Compared with the SWRO system, the produced water quality is improved.

#### 4.1.2. The BR-MSF and SWRO System with Independent Feed Water

The simulation results of the SWRO and BR-MSF system costs in option 2 are shown in Table 8 and Table 9. The total operating cost of the SWRO system is 7012.22 CNY, of which the operating energy consumption accounts for 65.13%. The unit price of SWRO system water production is 3.5061 CNY/m^3^. For the BR-MSF system, the total operating cost is 7397.14 CNY, of which the running energy consumption accounts for 26.81%. The unit price of BR-MSF water production is 7.3971 CNY/m^3^. Combining Table 7 and Table 9, it can be seen that the unit price of BR-MSF water production in option 2 is lower than that of the OT-MSF system in option 1. Therefore, the BR-MSF system is more energy efficient than the OT-MSF system to generate the same water resources.

It can be seen from Table 7 and Table 10 that to produce the same volume of fresh water, the feed water flowrate of the BR-MSF system is 34.7% less than that of the OT-MSF system. So, the BR-MSF saves more water than OT-MSF. Moreover, the option 2 also improves the water production ratio of the simple MSF system, reduces the water production price of the MSF system and improves the water production quality of the SWRO system. Comparing the overall water production of options 1 and 2, it can be found that the water production ratio of option 2 is higher than that of option 1, because the water production economy of BR-MSF system is better than that of the OT-MSF system.

#### 4.1.3. The Last-Stage Flash Water of the OT-MSF System as the Feed Water of the SWRO System

The cost simulation results of SWRO and OT-MSF systems in option 3 are shown in Table 11 and Table 12. Comparing Table 7 and Table 13, the water production cost of the SWRO and OT-MSF systems in option 3 is reduced by 120.3 m^3^/h and 92.66 m^3^/h. In options 1 and 3, the feed water flowrate of OT-MSF system is the same. In option 3, the feed water of the SWRO system is part of the wastewater from the OT-MSF system. Due to the high production temperature of the OT-MSF system, the permeation flux of the SWRO system is improved, so the feed water flowrate of the SWRO system of option 3 is lower than that of the SWRO system of option 1. In addition, option 3 reduces the wastewater management cost of the OT-MSF system. It can be seen from Table 13 that the blowdown temperature of OT-MSF system is 33.23 °C, which is higher than the independent feed seawater temperature of the original SWRO system. Therefore, the permeation flux of SWRO system decreases, the water production ratio is improved, and the water production cost of the whole system is reduced.

#### 4.1.4. The Last-Stage Flash Water of the BR-MSF System Is the Feed Water of the SWRO System

The cost-simulation results of SWRO and BR-MSF systems in option 4 are shown in Table 14 and Table 15. It is found from Table 10 and Table 16 that due to the use of the blowdown water with the heat of the BR-MSF system, the water production cost of SWRO and BR-MSF systems in option 4 is lower than that in option 2. It can be seen from Table 13 and Table 16 that the water production ratio of the BR-MSF system is higher than that of the OT-MSF system. Therefore, the water production ratio of option 4 is also higher than that of option 3, and since the BR-MSF system is more economical in water production, the water production cost of option 4 is also reduced compared to option 3. It also can be seen from the conclusion of option 2. Based on the above data, compared with the above three options, option 4 is the best structure with the lower operational cost.

#### 4.1.5. The Rejected Water of the BR-MSF System as the Feed Water of the SWRO System

In the option 5, rejected water in the BR-MSF system is used as the feed water of the SWRO system. The cost simulation results of SWRO and BR-MSF systems in option 5 are shown in Table 17 and Table 18. The total operating cost of the SWRO system is 6833.22 CNY, of which the operating energy consumption accounts for 65.84%. The unit price of SWRO system water production is 3.4166 CNY/m^3^. For the BR-MSF system, the total operating cost is 7287.39 CNY, of which the running energy consumption accounts for 27.21%. The unit price of BR-MSF system water production is 7.2874 CNY/m^3^.

According to Table 19, compared with the above four options, option 5 has the highest water production temperature, reaching 42.25 °C. Option 5 uses the rejected water of the BR-MSF system as the feed water of the SWRO system. This option increases the temperature of the feed water of the SWRO system and improves the seawater utilization rate of the BR-MSF system. So, the unit price of the BR-MSF system is lower than that of option 2. However, compared with option 4, the total water production price of option 5 is 70.28 CNY higher than that of option 4. So, option 5 has insufficient economic advantages.

#### 4.1.6. The Rejected Water of the SWRO System as Part of the Feed Water of the OT-MSF System

Differently from options 3, 4, and 5, in option 6, seawater and part of blowdown in the SWRO system are used as feed water of the OT-MSF system. The cost simulation results of SWRO and OT-MSF systems are shown in Table 20 and Table 21. The total operating cost of the SWRO system is 6964.63 CNY, of which the operating energy consumption accounts for 65.58%. The unit price of SWRO system water production is 3.4823 CNY/m^3^. For the OT-MSF system, the total operating cost is 7266.67 CNY, of which the running energy consumption accounts for 27.28%. The unit price of SWRO system water production is 7.2667 CNY/m^3^.

According to Table 19, option 6 uses the rejected water of the SWRO system as part of the feed water of the OT-MSF system, which saves the feed water of the OT-MSF system, comprehensively improves the quality of the water produced by the SWRO system, and reduces the amount of blowdown. Compared with option 2, the total price of water production in this option is reduced, and the water production ratio is reduced. This option is more economical than the option 2 system. This is because the water production by the SWRO system is reprocessed by the OT-MSF system, which reduces the cost of the entire system. However, compared with option 3, the total water production price of this option is higher. Because option 3 utilizes the waste heat of the OT-MSF system, the utilization rate of water resources is improved. Based on the comparison of other options, the advantages of option 6 are not outstanding.

#### 4.1.7. The Rejected Water of SWRO System as Part of the Feed Water of BR-MSF System

In option 7, the seawater and part of blowdown in the SWRO system are used as the feed water of BR-MSF system, which saves the feed water of BR-MSF system, comprehensively improves the water production quality of SWRO system and reduces the blowdown. The simulation results are shown in Table 22 and Table 23, and the water production of the hybrid system is shown in Table 24. According to comprehensive Table 24 and Table 25, the unit price of SWRO system water production in options 6 and 7 are the same, both being 3.4823 CNY/m^3^. Due to the economic advantages of the BR-MSF system in water production, option 7 has lower water production price than option 6, but compared with option 4, the economic advantages are insufficient. Because option 4 utilizes the waste heat of the BR-MSF system, the utilization rate of water resources is improved.

### 4.2. Evaluation of System Economics and Water Production Performance

#### 4.2.1. System Water Production Cost Evaluation

**Figure 10:**
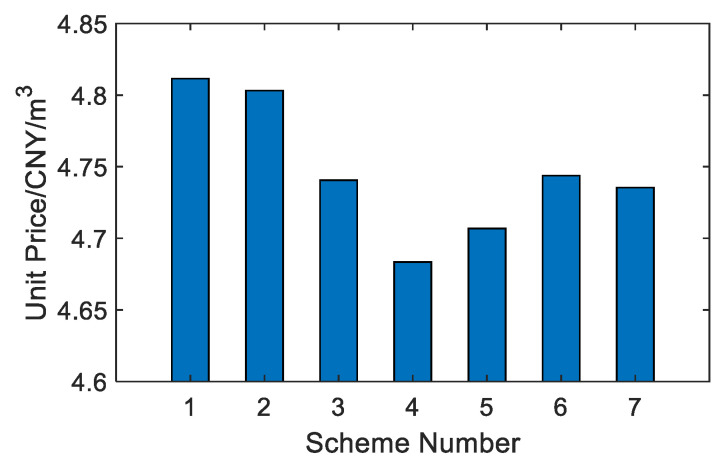
Comparison of unit price.

**Figure 11:**
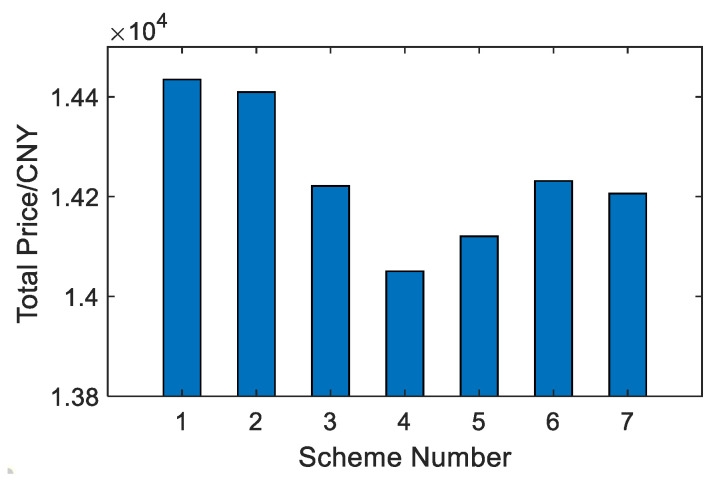
Comparison of total price.

As shown in Figure 10 and Figure 11, among the seven options, option 4 has the lowest water production price at 4.6834 CNY/m^3^, and option 1 has the highest water production price at 4.8115 CNY/m^3^. Option 4 is that the last-stage flash water from the BR-MSF system is used as the feed water of the SWRO system, which saves a lot of wastewater management costs of the BR-MSF system. Although the unit price advantage of system water production is not obvious, the total price of system water production is quite different.

#### 4.2.2. System Blowdown Flowrate Evaluation

**Figure 12:**
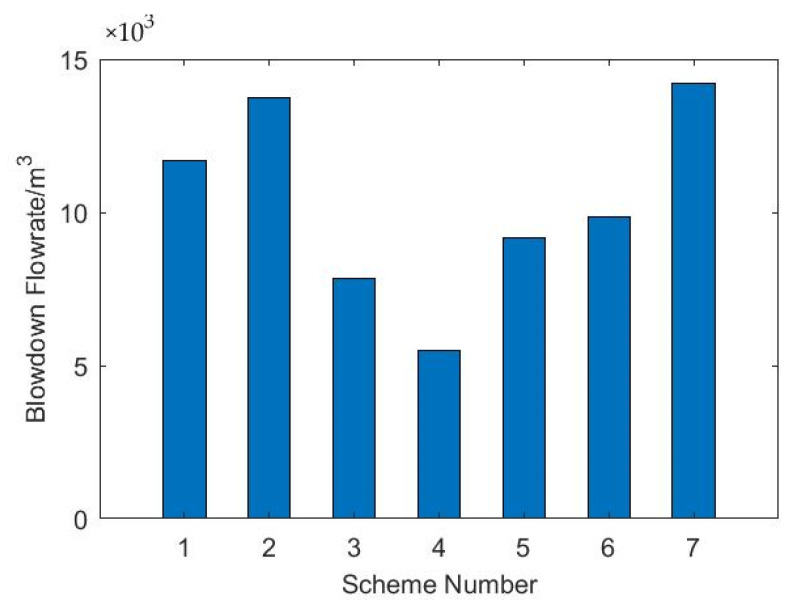
Comparison of blowdown flowrate.

As shown in Figure 12, among the seven options, option 4 has the lowest blowdown flowrate, with a blowdown flowrate of 5509.89 m^3^/h. Consistent with the comparison of system water prices, option 4 has an advantage in terms of system water price because of its blowdown flowrate.

#### 4.2.3. System Water Production Ratio Evaluation

**Figure 13:**
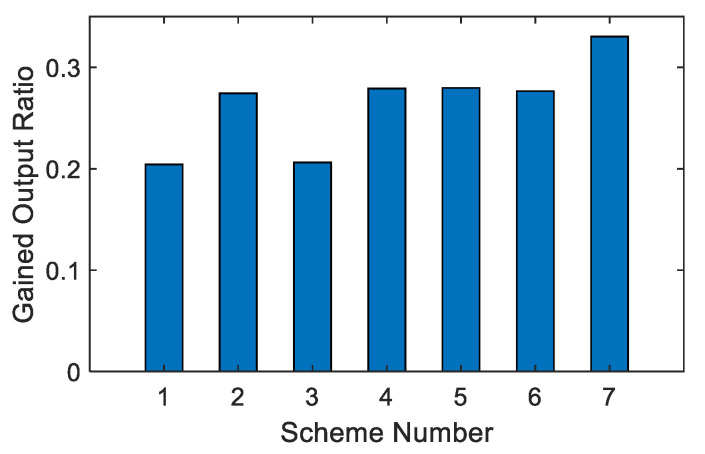
Comparison of water production ratio.

As shown in Figure 13, in the seven options, the water production ratio is almost the same, and the difference is not obvious. In comparison, option 7 has the highest water production ratio because all wastewater of the SWRO system flows into the BR-MSF system, which improves the overall water production ratio of the system. Comparing option 1 and option 2; option 3 and option 4; and option 6 and option 7—when the MSF hybrid system is the BR-MSF system, the corresponding option has more advantages in the comparison of water production ratio.

#### 4.2.4. System Water Production Temperature Evaluation

**Figure 14:**
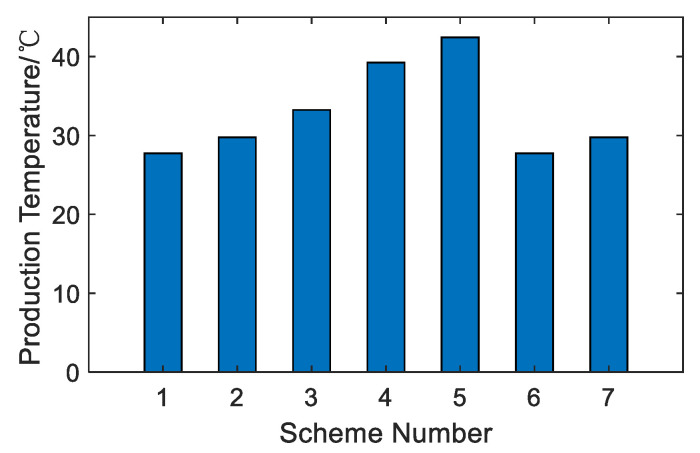
Comparison of production temperature.

As shown in Figure 14, among the seven options, option 5 has the highest water production temperature, followed by option 4. The water temperature of the SWRO system depends entirely on the influence of the feed temperature. In the option 5, the condensed water of the BR-MSF system in the coupling system is used as the feed water of the SWRO, which increases the water temperature of the SWRO system and comprehensively increases the water temperature of the entire option. In options 1, 2, 6, and 7, the temperature of the SWRO system is completely affected by the seawater temperature, so the water produced in the overall system is different from those in options 3, 4, and 5.

#### 4.2.5. Comprehensive Evaluation

As shown in Table 26, based on the systematic evaluation of the above four aspects, the seven hybrid options are comprehensively ranked in terms of water production price, blowdown flowrate, water production ratio, and production temperature. The four evaluation indexes are ranked by the advantages of low price, low flowrate, high water production ratio, and high temperature.

It can be seen from the results that option 4 ranks first among the seven options in terms of price and blowdown flowrate, and remains in the forefront in terms of water-making ratio. However, there is little difference between options 5 and 4 in terms of water-making ratio. Overall, option 4 has certain advantages and performs well in the four evaluation indexes. For the option 1 and option 2, they all rank last in the four evaluation indexes, with obvious disadvantages. The above results provide guidance for the feeding conditions of the MSF-RO system, and provide a certain basis for the subsequent optimization of system operation and the complexity of the hybrid option.

## 5. Conclusions

In addition to combining a new energy-driven system with RO or MSF seawater desalination to achieve better economic and social benefits, studies of better combinations of MSF-RO systems to obtain more techno-economic profits are also very attractive. In this work, based on the mechanism models of RO and MSF processes, modeling and structural optimization of hybrid MSF-RO system is carried out with the consideration of seven coupled structures. The feeding, discharging, and mixing separation modules of the system are modeled and the operational economics, the evaluation indexed are built. Simulation and calculation of the hybrid system with the seven coupled structures leading to the profiles of techno-economic performance as well as specific state values. The results show that: the fourth structural option has the lowest operational cost with the value of 4.6834 CNY/m^3^, which is obviously better than the other options. In addition, the fourth structural option can achieve the lowest wastewater blowdown. For minimizing the index of gained output ratio and production temperature, the seventh and the fifth option rank the first. More than that, from the numerical simulation and analysis, the evaluation index of the production price, blowdown flowrate, water production ratio, and water production temperature can all be obtained as well as those important state variables. Our work is helpful for the optimal operation and design of the MSF-RO system with detailed model-based numerical calculation. In the future work, more case studies for real plant application and the optimal control will be focused.

## Figures and Tables

**Table 1:** Water production of the SWRO system.

Parameters	Data
Feed water flowrate (m^3^/h)	12,030
Production flowrate (m^3^/h)	6255.24
Blowdown flowrate (kg/h)	5774.75
Number of pressure vessels	1204
Total number of RO modules	8428
Water production ratio	0.52

**Table 2:** SWRO system cost ratio table.

Cost	Total Price (CNY)	Unit Price (CNY/m^3^)	Proportion
*OC_IP_*	1185.31	0.1895	0.0540
*OC_CH_*	2827.05	0.4519	0.1289
*OC_EN_*	14,284.70	2.2836	0.6513
*OC_MER_*	1789.51	0.2861	0.0816
*OC_MN_*	657.94	0.1052	0.0300
*OC_LB_*_1_	1042.54	0.1667	0.0475
*OC_WS_*_1_	144.37	0.0231	0.0066
*OC_RO_*	21,931.41	3.5061	1

**Table 3:** Water production of the OT-MSF system.

Parameters	Data
Feed water flowrate (m^3^/h)	12,030
Production flowrate (m^3^/h)	1082.24
Heating steam mass flowrate (kg/h)	153,446.00
Blowdown flowrate (kg/h)	10,947.76
Gained output ratio	7.05
Water production ratio	0.09

**Table 4:** OT-MSF system cost ratio.

Cost	Total Price (CNY)	Unit Price (CNY/m^3^)	Proportion
*OC_ST_*	3662.41	3.3841	0.4435
*OC_EL_*	2188.14	2.0219	0.2649
*OC_MT_*	761.11	0.7033	0.0922
*OC_PR_*	393.71	0.3638	0.0477
*OC_LB_*_2_	979.76	0.9053	0.1186
*OC_WS_*_2_	273.69	0.2529	0.03314
*OC_MSF_*	8258.82	7.6313	1

**Table 5:** SWRO system cost ratio table.

Cost	Total Price (CNY)	Unit Price (CNY/m^3^)	Proportion
*OC_IP_*	378.98	0.1895	0.0540
*OC_CH_*	903.90	0.4519	0.1289
*OC_EN_*	4567.25	2.2836	0.6513
*OC_MER_*	572.23	0.2861	0.0816
*OC_MN_*	210.37	0.1052	0.0300
*OC_LB_*_1_	333.33	0.1667	0.04754
*OC_WS_*_1_	46.16	0.0231	0.0066
*OC_RO_*	7012.21	3.5061	1

**Table 6:** OT-MSF system cost ratio table.

Cost	Total Price (CNY)	Unit Price (CNY/m^3^)	Proportion
*OC_ST_*	3193.96	3.1940	0.4303
*OC_EL_*	2019.28	2.0193	0.2721
*OC_MT_*	703.27	0.7033	0.0948
*OC_PR_*	355.45	0.3555	0.0479
*OC_LB_*_2_	904.15	0.9042	0.1218
*OC_WS_*_2_	246.15	0.2461	0.0332
*OC_MSF_*	7422.27	7.4224	1

**Table 7:** Overall water production in option 1.

Parameters	SWRO	MSF	MSF-RO
Feed flowrate (m^3^/h)	3846.37	10,845.96	14,692.33
Production flowrate (m^3^/h)	2000	1000	3000
Blowdown flowrate (m^3^/h)	1846.38	9845.96	11,692.34
Water production ratio	0.52	0.09	0.20
Production concentration (kg/m^3^)	0.20	0	0.13
Production temperature (°C)	25	33.23	27.74
Total price (CNY)	7012.21	7422.27	14,434.48
Unit price (CNY/m^3^)	3.5061	7.4224	4.81

**Table 8:** SWRO system cost ratio table.

Cost	Total Price (CNY)	Unit Price (CNY/m^3^)	Proportion
*OC_IP_*	378.98	0.1895	0.0540
*OC_CH_*	903.90	0.4519	0.1289
*OC_EN_*	4567.25	2.2836	0.6513
*OC_MER_*	572.23	0.2861	0.0816
*OC_MN_*	210.37	0.1052	0.0300
*OC_LB_*_1_	333.33	0.1667	0.04754
*OC_WS_*_1_	46.16	0.0231	0.0066
*OC_RO_*	7012.22	3.5061	1

**Table 9:** BR-MSF system cost ratio table.

Cost	Total Price (CNY)	Unit Price (CNY/m^3^)	Proportion
*OC_ST_*	3285.80	3.2858	0.4442
*OC_EL_*	1983.25	1.9832	0.2681
*OC_MT_*	703.27	0.7033	0.0951
*OC_PR_*	239.30	0.2393	0.0324
*OC_LB_*_2_	888.02	0.8880	0.1200
*OC_WS_*_2_	297.50	0.2975	0.0402
*OC_MSF_*	7397.14	7.3971	1

**Table 10:** Overall water production in option 2.

Parameters	SWRO	MSF	MSF-RO
Feed water flowrate (m^3^/h)	3846.37	7083.82	10,930.19
Production flowrate (m^3^/h)	2000	1000	3000
Blowdown flowrate (m^3^/h)	1846.38	11,900	13,746.38
Water production ratio	0.52	0.14	0.27
Production concentration (kg/m^3^)	0.20	0	0.13
Production temperature (°C)	25	39.25	29.75
Total price (CNY)	7012.21	7397.14	14,409.35
Unit price (CNY/m^3^)	3.5061	7.3971	4.8031

**Table 11:** SWRO system cost ratio table.

Cost	Total Price (CNY)	Unit Price (CNY/m^3^)	Proportion
*OC_IP_*	365.19	0.1826	0.0530
*OC_CH_*	871.00	0.4355	0.1264
*OC_EN_*	4521.49	2.2607	0.6561
*OC_MER_*	551.42	0.2757	0.0801
*OC_MN_*	205.44	0.1027	0.0298
*OC_LB_*_1_	333.40	0.1667	0.0484
*OC_WS_*_1_	42.65	0.0213	0.0062
*OC_RO_*	6891.91	3.4460	1

**Table 12:** BR-MSF system cost ratio table.

Cost	Total Price (CNY)	Unit Price (CNY/m^3^)	Proportion
*OC_ST_*	3193.96	3.1939	0.4358
*OC_EL_*	2019.28	2.0193	0.2755
*OC_MT_*	703.27	0.7033	0.0959
*OC_PR_*	355.45	0.3555	0.0485
*OC_LB_*_2_	904.15	0.9042	0.1234
*OC_WS_*_2_	153.49	0.1535	0.0209
*OC_MSF_*	7329.61	7.3296	1

**Table 13:** Overall water production in option 3.

Parameters	SWRO	MSF	MSF-RO
Feed flowrate (m^3^/h)	3706.40	10,845.96	14,552.36
Production flowrate (m^3^/h)	2000	1000	3000
Blowdown flowrate (m^3^/h)	1706.02	6139.56	7845.58
Water production ratio	0.54	0.09	0.21
Production concentration (kg/m^3^)	0.20	0	0.13
Production temperature (°C)	33.23	33.23	33.23
Total price (CNY)	6891.91	7329.61	14,221.52
Unit price (CNY/m^3^)	3.4460	7.3296	4.7405

**Table 14:** SWRO system cost ratio table.

Cost	Total Price (CNY)	Unit Price (CNY/m^3^)	Proportion
*OC_IP_*	378.98	0.1895	0.0544
*OC_CH_*	903.90	0.4519	0.1298
*OC_EN_*	4567.25	2.2836	0.6558
*OC_MER_*	572.23	0.2861	0.0822
*OC_MN_*	208.94	0.1045	0.0300
*OC_LB_*_1_	333.33	0.1667	0.0479
*OC_WS_*_1_	0	0	0
*OC_RO_*	6854.52	3.4273	1

**Table 15:** BR-MSF system cost ratio table.

Cost	Total Price (CNY)	Unit Price (CNY/m^3^)	Proportion
*OC_ST_*	3285.80	3.2858	0.4566
*OC_EL_*	1983.25	1.9832	0.2756
*OC_MT_*	703.27	0.7033	0.0977
*OC_PR_*	239.30	0.2393	0.0333
*OC_LB_*_2_	888.02	0.8880	0.1234
*OC_WS_*_2_	96.17	0.0962	0.0134
*OC_MSF_*	7195.81	7.1958	1

**Table 16:** Overall water production in option 4.

Parameters	SWRO	MSF	MSF-RO
Feed water flowrate (m^3^/h)	3663.14	7083.82	10,746.95
Production flowrate (m^3^/h)	2000	1000	3000
Blowdown flowrate (m^3^/h)	1663.03	3846.86	5509.89
Water production ratio	0.55	0.14	0.28
Production concentration (kg/m^3^)	0.20	0	0.13
Production temperature (°C)	39.25	39.25	39.25
Total price (CNY)	6854.52	7195.81	14,050.33
Unit price (CNY/m^3^)	3.4273	7.1958	4.6834

**Table 17:** SWRO system cost ratio table.

Cost	Total Price (CNY)	Unit Price (CNY/m^3^)	Proportion
*OC_IP_*	358.64	0.1793	0.0525
*OC_CH_*	855.38	0.4277	0.1252
*OC_EN_*	4498.86	2.2494	0.6584
*OC_MER_*	541.01	0.2705	0.0792
*OC_MN_*	205.00	0.1025	0.0300
*OC_LB_*_1_	333.33	0.1667	0.0488
*OC_WS_*_1_	41.00	0.0205	0.0060
*OC_RO_*	6833.22	3.4166	1

**Table 18:** BR-MSF system cost ratio table.

Cost	Total Price (CNY)	Unit Price (CNY/m^3^)	Proportion
*OC_ST_*	3285.80	3.2858	0.4509
*OC_EL_*	1983.25	1.9832	0.2721
*OC_MT_*	703.27	0.7033	0.0965
*OC_PR_*	239.30	0.2393	0.0328
*OC_LB_*_2_	888.02	0.8880	0.1219
*OC_WS_*_2_	187.75	0.1878	0.0258
*OC_MSF_*	7287.39	7.2874	1

**Table 19:** Overall water production in option 5.

Parameters	SWRO	MSF	MSF-RO
Feed flowrate (m^3^/h)	3639.91	7083.81	10,723.73
Production flowrate (m^3^/h)	2000	1000	3000
Blowdown flowrate (m^3^/h)	1639.91	7510	9149.91
Water production ratio	0.55	0.14	0.28
Production concentration (kg/m^3^)	0.20	0	0.13
Production temperature (°C)	44.96	37.43	42.45
Total price (CNY)	6833.22	7287.39	14,120.61
Unit price (CNY/m^3^)	3.4166	7.2874	4.7069

**Table 20:** SWRO system cost ratio table.

Cost	Total Price (CNY)	Unit Price (CNY/m^3^)	Proportion
*OC_IP_*	378.98	0.1895	0.0544
*OC_CH_*	903.90	0.4519	0.1298
*OC_EN_*	4567.25	2.2836	0.6558
*OC_MER_*	572.23	0.2861	0.0822
*OC_MN_*	208.94	0.1045	0.0300
*OC_LB_*_1_	333.33	0.1667	0.0479
*OC_WS_*_1_	0	0	0
*OC_RO_*	6964.63	3.4823	1

**Table 21:** OT-MSF system cost ratio table.

Cost	Total Price (CNY)	Unit Price (CNY/m^3^)	Proportion
*OC_ST_*	3193.96	3.1940	0.4395
*OC_EL_*	1982.44	1.9824	0.2728
*OC_MT_*	703.27	0.7033	0.0968
*OC_PR_*	236.70	0.2367	0.0326
*OC_LB_*_2_	904.15	0.9042	0.1244
*OC_WS_*_2_	246.15	0.2461	0.0339
*OC_MSF_*	7266.67	7.2667	1

**Table 22:** SWRO system cost ratio table.

Cost	Total Price (CNY)	Unit Price (CNY/m^3^)	Proportion
*OC_IP_*	378.98	0.1895	0.0544
*OC_CH_*	903.90	0.4519	0.1298
*OC_EN_*	4567.25	2.2836	0.6558
*OC_MER_*	572.23	0.2861	0.0822
*OC_MN_*	208.94	0.1045	0.0300
*OC_LB_*_1_	333.33	0.1667	0.0479
*OC_WS_*_1_	0	0	0
*OC_RO_*	6964.63	3.4823	1

**Table 23:** OT-MSF system cost ratio table.

Cost	Total Price (CNY)	Unit Price (CNY/m^3^)	Proportion
*OC_ST_*	3285.80	3.2858	0.4537
*OC_EL_*	1946.41	1.9464	0.2688
*OC_MT_*	703.27	0.7033	0.0971
*OC_PR_*	120.54	0.1205	0.0166
*OC_LB_*_2_	888.02	0.8880	0.1226
*OC_WS_*_2_	297.50	0.2975	0.0411
*OC_MSF_*	7241.54	7.2415	1

**Table 24:** Overall water production in option 4.

Parameters	SWRO	MSF	MSF-RO
Feed water flowrate (m^3^/h)	3846.37	5237.43	9083.81
Production flowrate (m^3^/h)	2000	1000	3000
Blowdown flowrate (m^3^/h)	0	11,900	11,900
Water production ratio	0.52	0.14	0.33
Production concentration (kg/m^3^)	0.20	0	0.13
Production temperature (°C)	25	39.25	29.75
Total price (CNY)	6964.63	7241.54	14,206.17
Unit price (CNY/m^3^)	3.4823	7.2415	4.7354

**Table 25:** Overall water production in option 6.

Parameters	SWRO	MSF	MSF-RO
Feed water flowrate (m^3^/h)	3846.37	6999.59	10,845.96
Production flowrate (m^3^/h)	2000	1000	3000
Blowdown flowrate (m^3^/h)	0	9845.96	9845.96
Water production ratio	0.52	0.09	0.28
Production concentration (kg/m^3^)	0.20	0	0.13
Production temperature (°C)	25	33.23	27.74
Total price (CNY)	6964.63	7266.67	14,231.30
Unit price (CNY/m^3^)	3.4823	7.2667	4.7438

**Table 26:** Number of variables with IDM.

	Rankin	No. 1	No. 2	No. 3	No. 4	No. 5	No. 6	No. 7
Index								
Price		Option 4	Option 5	Option 7	Option 3	Option 6	Option 2	Option 1
Blowdown		Option 4	Option 3	Option 5	Option 6	Option 1	Option 7	Option 2
Gained output ratio		Option 7	Option 5	Option 4	Option 6	Option 2	Option 3	Option 1
Production temperature		Option 5	Option 4	Option 3	Option 7	Option 2	Option 6	Option 1

## Data Availability

Not applicable.

## Abbreviations

*a*	decay level of water permeability
*A_w_*	membrane water permeability (m·s^−1^·Pa^−1^)
*A*_*w*0_	intrinsic membrane water permeability (m·s^−1^·Pa^−1^)
*b*	decay level of TDS permeability
*B_s_*	membrane TDS permeability (m/s)
*B*_*s*0_	intrinsic membrane TDS permeability (m/s)
*C_b_*	bulk concentration along feed channel (kg/m^3^)
*C_f_*	feed salt concentration (kg/m^3^)
*C_m_*	salt concentration of membrane surface (kg/m^3^)
*C_p_*	permeate concentration of RO unit (kg/m^3^)
*C_r_*	brine concentration (kg/m^3^)
*C_sp_*	permeate concentration of permeate side (kg/m^3^)
*FA*	irreversible degradation coefficient of *A_w_*
*FB*	irreversible degradation coefficient of *B_s_*
*Jv*	solvent flux (kg/m^2^·s)
*Js*	solute flux (kg/m^2^·s)
*L*	length of the RO channel (m)
*MFA*	membrane performance coefficient of *A_w_*
*MFB*	membrane performance coefficient of *B_s_*
*MOD*	number of membrane module
*Ncl*	cleaning frequency
*n*_l_	number of leaves in RO module
*n_pv_*	number of pressure vessel
*OC*	operational cost of whole process
*OC_EN_*	energy cost of RO process
*OC_BP_*	energy cost of boost pump
*OC_CH_*	cost for chemicals
*OC_IP_*	energy cost for intake and pretreatment
*OC_ME_*	cost for membrane replacement
*OC_MN_*	cost for system maintenance
*OC_MNCON_*	cost for conventional equipment maintenance
*OC_MNCL_*	cost for membrane module cleaning
*OC_OT_*	membrane cleaning cost for chemicals
*OC_PC_*	membrane cleaning cost for other
*P_b_*	pressure along feed channel (bar)
*P_elc_*	electricity price
*P_d_*	pressure drop along RO spiral wound module (bar)
*P_f_*	feed pressure (bar)
*P_p_*	pressure in permeate side (bar)
*P_r_*	brine pressure (bar)
*P_ri_*	membrane price
*PLF*	load coefficient
*Q_f_*	feed flowrate (m^3^/h)
*Q_p_*	permeate flowrate (m^3^/h)
*Q_r_*	brine flowrate (m^3^/h)
*WR*	water recovery ratio
*SR*	salt rejection coefficient (%)
*SEC*	specific energy consumption (kw·h/m^3^)
*t*	operation time of membrane module (day)
*t_q_*	membrane operation time after last cleaning
*T*	feed (operational) temperature (K)
*V_b_*	axial velocity of bulk flow (m/s)
*V_f_*	axial velocity of feed flow (m/s)
*V_r_*	axial velocity of brine flow (m/s)
*W*	width of the RO module (m)
*Xcl*	membrane cleaning time
*Xmr*	membrane replacing time
*α*_1_, *α*_2_, *β*_1_	constant parameters
*∆π*	pressure loss of osmosis pressure (bar)
*∆T*	time of each schedule period
*∆Tcl*	membrane use time since last cleaning
*ρ*	density of permeate water (kg/m^3^)
*λ*	friction factor
*ζ*	membrane replacing rate
*ε_p_*	mechanical efficiency of high-pressure pump
*ε_pf_*	energy recovery efficiency
*b*	bulk
*f*	module feed channel
*m*	membrane surface
*p*	permeate side
*r*	brine side
